# Factors associated with single versus multiple supernumerary teeth in a paediatric population: a cross-sectional study

**DOI:** 10.1007/s40368-026-01171-5

**Published:** 2026-03-12

**Authors:** C. de O. Gomes, L. B. Nogueira, P. C. Goulart, V. Z. Drumond, J. A. A. de Arruda, L. G. Abreu, M. L. Ramos-Jorge, M. H. N. G. Abreu, R. A. Mesquita

**Affiliations:** School of dentistry, Universidade Federal de Minas Gerais, Belo Horizonte, Brazil

**Keywords:** Hyperdontia, Paediatric dentistry, Supernumerary teeth, Supplemental tooth

## Abstract

**Purpose:**

Supernumerary teeth (ST) are developmental anomalies. However, factors associated with ST have not been extensively reported in the literature. The aim of the present study was to identify factors associated with single supernumerary teeth (SST) and multiple supernumerary teeth (MST) in a non-syndromic Brazilian sample.

**Methods:**

A cross-sectional study was conducted with a convenience sample of 305 non-syndromic patients with ST seen over a 10-year period at a paediatric oral surgery service in Southeast Brazil. The outcome variable was the classification of ST as SST or MST. Covariates included age, race, sex, morphology, location, position, eruption status, orientation, associated complications, and treatment modality of ST. The Mann–Whitney test, chi-squared test, and both simple and multiple logistic regression analyses were performed.

**Results:**

A total of 460 ST were identified among the 305 patients, with male predominance (68.2%). Mean age was 9.3 years. Most patients had SST (62.2%) and 37.8% had MST. ST were primarily located in the maxilla (93.1%), with fewer cases in the mandible or both arches. MST were strongly associated with specific position, eruption, orientation, clinical complication, morphological, and treatment characteristics (*p* < 0.001).

**Conclusion:**

Tuberculate and supplemental ST, palatal positioning, and eruption status were significantly associated with an increased likelihood of MST. Moreover, MST were associated with a greater risk of adjacent tooth displacement and greater need for combined orthodontic treatment and extraction.

## Introduction

Supernumerary teeth (ST) constitute a complex dental anomaly with a multifactorial aetiology influenced by both genetic and environmental factors (Davidson et al. 2025). This is a well-documented clinical phenomenon defined as teeth that exceed the normal complement of the dentition, which may be erupted or unerupted (Talaat et al. 2022). The prevalence of ST ranges from 0.1 to 3.8% in the permanent dentition, whereas occurrence in the primary dentition is rare (Cassetta et al. 2014; Katanaki et al. 2025; Maddalone et al. 2018). This condition is generally more frequent in males, with a male-to-female ratio of approximately 2.5:1, and the maxilla is the most commonly affected site (Cassetta et al. 2014; Davidson et al. 2025; Henninger et al. 2023). However, a recent South African study reported a 2.5% rate of ST, with no difference between the sexes (Thomas et al. 2023). ST can give rise to several clinical complications ranging from simpler conditions, such as tooth crowding and diastema, to more complex conditions, including dentigerous cysts (Adhikari et al. 2025; Anthonappa et al. 2013).

ST may occur as single or multiple teeth, unilaterally or bilaterally in the maxilla, mandible, or both jaws (Davidson et al. 2025; Ma et al. 2021). Multiple supernumerary teeth (MST) are most commonly associated with syndromes (e.g., cleidocranial dysplasia, familial adenomatous polyposis, and trichorhinophalangeal syndrome type I) (Lubinsky and Kantaputra 2016). Moreover, individuals with non-syndromic cleft lip and/or palate are more likely to exhibit MST (Fonseca-Souza et al. 2022), particularly in European populations (Azevedo et al. 2025).

The management of ST may involve surgical removal, combined or not with orthodontic treatment, or follow-up alone, with the decision-making process guided by clinical and radiographic findings (Davidson et al. 2025; Ma et al. 2021). Management strategies are determined by the type, position, and potential impact of ST on adjacent structures. The aim of early intervention is to prevent or minimise associated complications (Seehra et al. 2023). Surgical removal, particularly in the mixed dentition period, is often recommended to facilitate the eruption of permanent teeth and support normal alignment. In select cases, however, an observation period may be appropriate, depending on the individual clinical situation (Cortés-Bretón-Brinkmann et al. 2019; Seehra et al. 2023).

Most studies on ST address clinical and demographic characteristics, including mean age, sex, ethnicity, anatomical location, and tooth position (Cassetta et al. 2014; Ma et al. 2021; Thomas et al. 2023). The aim of the present study was to investigate clinical variables more associated with single supernumerary teeth (SST) or MST in a non-syndromic population.

## Materials and methods

### Study design and ethical aspects

This investigation was designed as a cross-sectional study and is reported in accordance with the Strengthening the Reporting of Observational Studies in Epidemiology (STROBE) guidelines (von Elm et al. 2008). Prior to data collection, the study protocol received approval from the Human Ethics Committee of *Universidade Federal de Minas Gerais* (UFMG; approval number 363/05).

### Sample and setting

This study involved a convenience sample of 305 non-syndromic Brazilian children four to 16 years of age with ST in different regions of the jaws. Patients were screened at the UFMG paediatric oral surgery service between 1995 and 2004. All consecutively assessed non-syndromic patients with ST during this period were included in the study.

### Variables and data collection

The sociodemographic variables of interest were age (years) and sex. Clinical variables comprised the presence of complications associated with ST (yes/no), including the delayed eruption of adjacent teeth, displacement, diastema, rotation, and root resorption. Morphological characteristics were classified as supplemental, conical, or tuberculate. Positional variables included tooth orientation (normal, inverted, inclined, or horizontal) and sagittal position (labial/buccal, palatal/lingual, or within the dental arch). The anatomical site was defined according to jaw and tooth region, encompassing maxillary and mandibular locations. Developmental stage was categorised as crown under formation, fully developed crown, fully developed crown with root under formation, or fully developed tooth. Management-related variables included treatment modality (surgical removal alone or surgical removal combined with orthodontic treatment) and the performance of clinical follow-up.

### Outcome assessment

The outcome variable (SST or MST) was assessed by a single experienced examiner who had undergone a two-phase process to ensure intra-rater reliability. Agreement between the two assessment periods was determined using the Kappa coefficient (*κ* = 0.85). The diagnosis of ST was established through a clinical examination and the analysis of occlusal, periapical, or panoramic radiographs. MST was defined when patients had more than one ST. Odontomas were not considered in this study, as there is no global consensus regarding their classification as ST (Garvey et al. 1999). The main independent variables considered were sex and age, clinical complications associated with ST, and the morphology, orientation, sagittal position, location, developmental stage, and management of ST. The characteristics of ST were confirmed at the time of surgical removal.

### Statistical analysis

The results were organised and entered into a dataset using the Statistical Package for the Social Sciences (SPSS, IBM Corp. Armonk, NY, USA), version 27.0. Statistical analysis initially involved descriptive statistics, with absolute and relative frequencies calculated for the variables of interest. Associations between independent variables and the outcome were assessed using bivariate analysis (chi-squared test). The absence of an association between variables (*p* > 0.05) was considered the null hypothesis. Variables eligible for inclusion in the multiple logistic regression model were identified based on the results of the bivariate analysis. Variables with a *p*-value < 0.10 were included in the final multiple logistic regression analysis. Variables were entered into the model in increasing order of statistical significance and were retained only if remaining statistically significant. To determine independent associations with the presence of MST, the variables were included one at a time into an unconditional multiple logistic regression model. The final model was adjusted for the effect of all variables, including sex, and the effect of each exposure on the likelihood of the presence of two or more ST was estimated. Adjusted odds ratios (OR) and 95% confidence intervals (CI) were calculated for each variable included in the logistic regression model.

## Results

### Sample characteristics

**Fig. 1:**
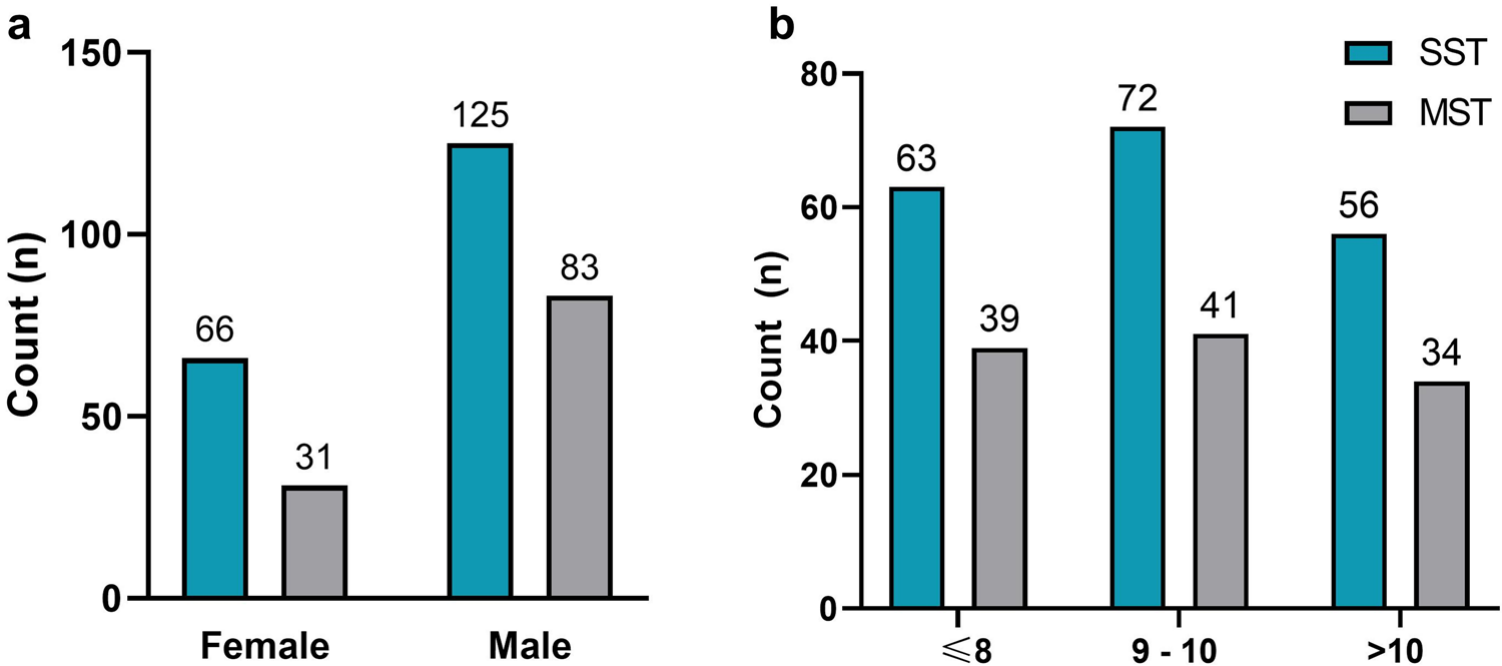
Plot showing occurrence of single or multiple supernumerary teeth according to sex (**a**) and age (**b**)

**Fig. 2:**
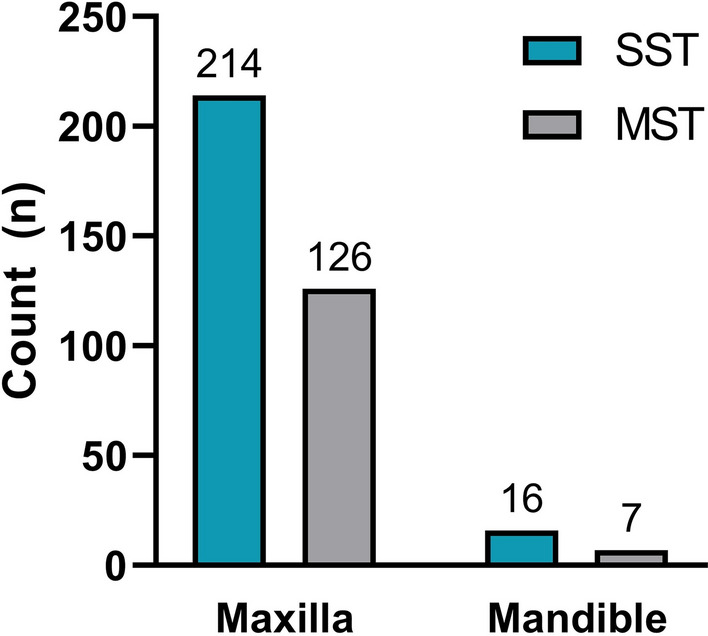
Plot showing occurrence of single or multiple supernumerary teeth in maxilla and mandible

Boys predominated in the sample (68.2%) (Fig. 1a). Most children were 10 years of age or younger (mean: 9.3 ± 2.3 years) (Fig. 1b). A total of 460 ST were identified among the 305 patients, yielding a mean of 1.51 ± 0.9 ST per individual. Boys accounted for 318 ST, whereas girls accounted for 143 ST, resulting in a male-to-female ratio of 2.2:1, with a mean of 1.53 ± 0.9 and 1.46 ± 1.0 ST per patient, respectively. A total of 190 (62.2%) patients had one SST, 97 (31.8%) had two ST, and the remaining 18 (6.0%) had between three and nine ST. The majority of children (*n* = 284; 93.1%) had ST exclusively in the maxilla, whereas 12 (3.9%) had ST only in the mandible, and nine (3.0%) had ST in both arches (Fig. 2).

### Bivariate analysis

The bivariate analysis (chi-squared test) revealed statistically significant associations between SST or MST and the following variables: complications (delayed eruption and displacement), eruption status, morphology (supplemental and tuberculate), normal orientation, sagittal position (lingual, palatal, or within the arch), developmental stage (crown under formation and fully developed crown), and treatment option (orthodontic treatment combined with extraction and follow-up alone) (Table 1).

**Table 1:** Factors associated to single or multiple supernumerary teeth (Pediatric Oral Surgery Service of the Federal University of Minas Gerais)

Variables	Supernumerary teeth			*p*-value
	Single *n* (%)	Multiple *n* (%)	Total *n* (%)	
Gender				
Female	66 (68.0)	31 (32.0)	97 (100)	0.182*
Male	125 (60.1)	83 (39.9)	208 (100)	
Age				
≤ 8 years	63 (61.8)	39 (38.2)	102 (100)	0.953*
9 and 10 years	72 (63.7)	41 (36.3)	113 (100)	
> 10 years	56 (62.2)	34 (37.8)	90 (100)	
Clinical complications				
No	30 (85.7)	5 (14.3)	35 (100)	0.003*
Yes	161 (59.6)	109 (40.4)	270 (100)	
Delayed eruption				
No	104 (70.3)	44 (29.7)	148 (100)	0.007*
Yes	87 (55.4)	70 (44.6)	157 (100)	
Displacement				
No	94 (72.3)	36 (27.7)	130 (100)	0.003*
Yes	97 (55.4)	78 (44.6)	175 (100)	
Diastema				
No	145 (64.2)	81 (35.8)	226 (100)	0.348*
Yes	46 (58.2)	33 (41.8)	79 (100)	
Rotation				
No	163 (63.7)	93 (36.3)	256 (100)	0.387*
Yes	28 (57.1)	21 (42.9)	49 (100)	
Resorption				
No	190 (62.7)	13 (37.3)	303 (100)	1.000^†^
Yes	1 (50.0)	1 (50.0)	2 (100)	
Eruption				
Unerupted	148 (68.8)	67 (31.2)	215 (100)	0.001*
One or more erupted	43 (47.8)	47 (52.2)	90 (100)	
Morphology				
Supplemental				
No	169 (66.0)	87 (34.0)	256 (100)	0.005*
Yes	22 (44.9)	27 (55.1)	49 (100)	
Conical				
No	83 (61.0)	53 (39.0)	136 (100)	0.606*
Yes	108 (63.9)	61 (36.1)	169 (100)	
Tuberculate				
No	131 (74.4)	45 (25.6)	176 (100)	< 0.001*
Yes	60 (46.5)	69 (53.5)	129 (100)	
Orientation				
Normal				
No	63 (95.5)	3 (4.5)	66 (100)	< 0.001*
Yes	127 (53.4)	111 (46.6)	238 (100)	
Inverted				
No	140 (61.4)	88 (38.6)	228 (100)	0.449*
Yes	51 (66.2)	26 (33.8)	77 (100)	
Inclined				
No	181 (62.4)	109 (37.6)	290 (100)	0.740*
Yes	10 (66.7)	5 (33.3)	15 (100)	
Horizontal				
No	189 (62.6)	113 (37.4)	302 (100)	1.000^†^
Yes	2 (66.7)	1 (33.3)	3 (100)	
Sagittal position				
Labial/Buccal				
No	176 (61.3)	111 (38.7)	287 (100)	0.061*
Yes	15 (83.3)	3 (17.7)	18 (100)	
Palatal/lingual				
No	34 (82.9)	7 (17.1)	41 (100)	0.004*
Yes	157 (59.5)	107 (40.5)	264 (100)	
Within arch				
No	173 (65.8)	90 (34.2)	263 (100)	0.004*
Yes	18 (42.9)	24 (57.1)	42 (100)	
Anatomical site				
Maxillary midline				
No	117 (63.6)	67 (36.4)	184 (100)	0.993*
Yes	77 (63.6)	44 (36.4)	121 (100)	
Maxillary central incisor				
No	88 (62.9)	52 (37.1)	140 (100)	0.802*
Yes	106 (64.2)	59 (35.8)	165 (100)	
Maxillary lateral incisor				
No	172 (63.7)	98 (36.3)	270 (100)	0.922*
Yes	22 (62.9)	13 (37.1)	35 (100)	
Maxillary canine				
No	189 (64.3)	105 (35.7)	294 (100)	0.216^†^
Yes	5 (45.5)	6 (54.5)	11 (100)	
Maxillary premolar				
No	191 (64.1)	107 (35.9)	298 (100)	0.262^†^
Yes	3 (42.9)	4 (57.1)	7 (100)	
Maxillary molar				
No	193 (63.5)	111 (36.5)	304 (100)	1.000^†^
Yes	1 (100)	0 (0.0)	0 (100)	
Mandibular central incisor				
No	194 (63.8)	110 (36.2)	304 (100)	0.364^†^
Yes	0 (0.0)	1 (100)	1 (100)	
Mandibular lateral incisor				
No	191 (63.2)	111 (36.8)	302 (100)	0.556^†^
Yes	3 (100)	0 (0.0)	3 (100)	
Mandibular canine				
No	190 (63.8)	108 (36.2)	298 (100)	1.000^†^
Yes	4 (66.7)	2 (33.3)	6 (100)	
Mandibular premolar				
No	185 (63.4)	107 (36.6)	292 (100)	0.775^†^
Yes	9 (69.2)	4 (30.8)	13 (100)	
Developmental stage				
Crown under formation				
No	190 (65.3)	101 (34.7)	291 (100)	0.005*
Yes	4 (28.6)	10 (71.4)	14 (100)	
Fully developed crown				
No	163 (68.8)	74 (31.2)	237 (100)	< 0.001*
Yes	31 (45.6)	37 (54.4)	68 (100)	
Fully developed crown with root under formation				
No	124 (64.6)	68 (35.4)	192 (100)	0.644*
Yes	70 (61.9)	43 (38.1)	113 (100)	
Fully developed tooth				
No	105 (67.7)	50 (32.3)	155 (100)	0.127*
Yes	89 (59.3)	61 (40.7)	150 (100)	
Treatment options				
Surgical removal and orthodontic treatment				
No	92 (79.3)	24 (20.7)	116 (100)	< 0.001*
Yes	99 (52.4)	90 (47.6)	189 (100)	
Surgical removal				
No	135 (59.5)	92 (40.5)	227 (100)	0.052*
Yes	56 (71.8)	22 (28.2)	78 (100)	
Clinical follow-up				
No	155 (58.1)	112 (41.9)	267 (100)	< 0.001*
Yes	36 (94.7)	2 (5.3)	38 (100)	

^*^Pearson chi-squared tests

^†^Fisher exact tests

### Multiple logistic regression

**Fig. 3:**
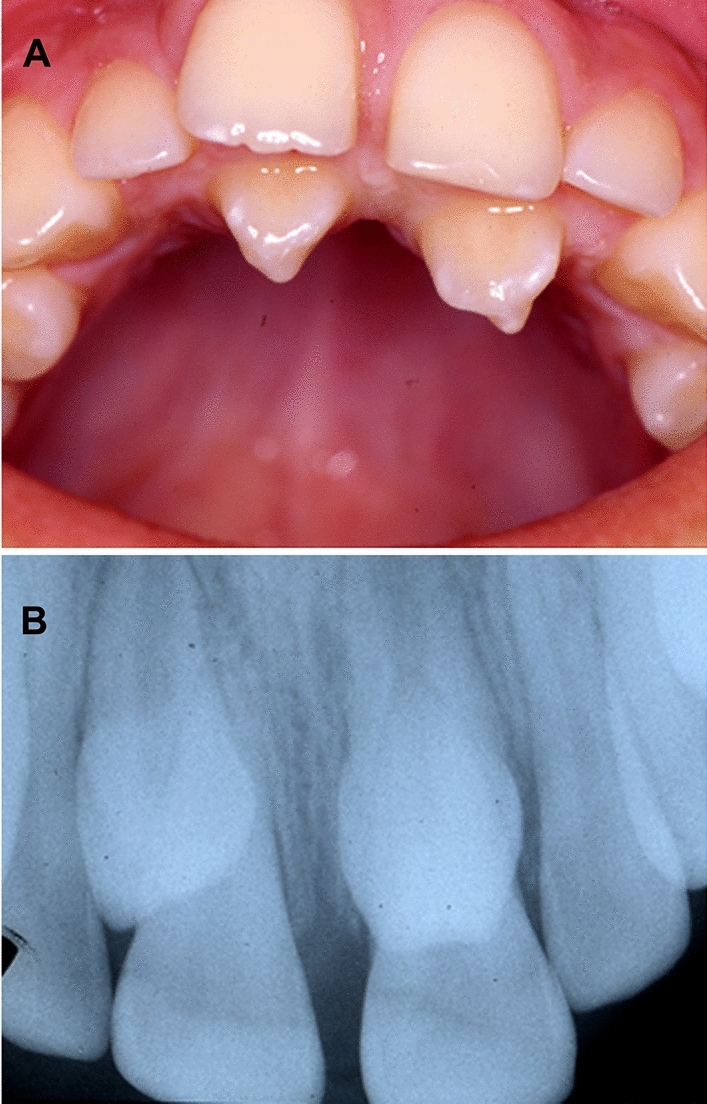
Clinical (**a**) and radiographic (**b**) features of erupted, supplemental multiple supernumerary teeth in palatal position

Multiple logistic regression analysis was performed to identify variables independently associated with the presence of two or more ST (Table 2). Patients with tuberculate (OR = 3.21; *p* = 0.001) or supplemental (OR = 2.28; *p* = 0.045) ST were more likely to have MST. ST located in a palatal position (OR = 9.97; *p* = 0.001) (Fig. 3) significantly increased the likelihood of MST compared to those located in a buccal or within-arch position. Individuals with at least one erupted ST were 3.78 times more likely (*p* = 0.001) to have MST than those without erupted ST (Fig. 3). Furthermore, the presence of MST increased the likelihood of adjacent tooth displacement (OR = 2.75; *p* = 0.001). Orientation also played a role, as normally oriented ST were associated with an 8.04-fold greater likelihood (*p* = 0.001) of MST compared to inverted or transversely oriented ST. Lastly, individuals with MST had a 2.22-fold greater likelihood (*p* = 0.022) of undergoing combined orthodontic treatment and extraction compared to extraction alone or simple follow-up.

**Table 2:** Simple and multiple logistic regression analysis between independent variables and single or multiple supernumerary teeth (final model)

Variable	Unadjusted odds ratio (CI 95%)	*p*-value	Adjusted odds ratio (CI 95%)	*p*-value*
Sagittal position				
Lingual	1	0.006	1	< 0.001
Palatal	3.31 (1.4–7.7)		9.97 (3.5–27.9)	
Eruption of ST				
Unerupted	1	0.001	1	< 0.001
One or more erupted	2.41 (1.4–3.9)		3.78 (1.8–7.7)	
Orientation normal				
No	1	< 0.001	1	0.001
Yes	18.35 (5.6–60.1)		8.04 (2.2–29.1)	
Displacement due ST				
No	1	0.003	1	0.001
Yes	2.10 (1.3–3.4)		2.75 (1.5–5.1)	
Type tuberculate				
No	1	< 0.001	1	0.001
Yes	3.35 (2.1–5.4)		3.21 (1.6–6.5)	
Type supplemental				
No	1	0.006	1	0.045
Yes	2.38 (1.3–4.4)		2.28 (1.1–5.1)	
Orthodontic treatment and extraction				
No	1	< 0.001	1	0.022
Yes	3.48 (2.0–5.9)		2.22 (1.1–4.4)	

*ST* supranumary teeth

^*^Adjusted for gender

## Discussion

The findings of this cross-sectional study indicate that ST with tuberculate and supplemental morphology, palatal positioning, at least one erupted ST, and normal orientation were more frequently associated with MST. Another important observation regards complications and treatment approaches, as individuals with MST were more likely to experience displacement caused by ST and were more commonly managed with a combination of surgical intervention and orthodontic treatment. Collectively, these characteristics may serve as potential indicators of the presence of MST, demonstrating their relevance in clinical assessments and the planning of treatment.

Findings from previous studies on ST appear to vary according to the population analysed. Some studies reported that the prevalence was higher among boys (Cassetta et al. 2014; Henninger et al. 2023), while others found that girls were more frequently affected (Singh et al. 2014). Despite this variability, the anatomical distribution of ST is relatively consistent across studies, with a marked predominance in the maxilla (Anthonappa et al. 2013; Davidson et al. 2025; Ma et al. 2021). In the present study, a significant difference was found between anatomical sites, with the maxilla disproportionately affected, especially among males. Although the biological basis underpinning these patterns is not fully understood, current evidence suggests that the development of ST is driven by heterogeneous and possibly overlapping mechanisms rather than a single pathogenic pathway (Anthonappa et al. 2013; Cammarata-Scalisi et al. 2018). The results of previous studies lend support to the involvement of dysregulated dental lamina activity, potentially mediated by alterations in key odontogenic signalling pathways, including WNT/β-catenin, Sonic hedgehog, and bone morphogenetic proteins, which regulate the number, patterning, and morphogenesis of teeth (Kyeong et al. 2025; Liu et al. 2008; Sagai et al. 2017). Variability in the activation or timing of these pathways may partially explain differences in anatomical distribution, sex predilection, and the occurrence of SST versus MST. In this context, population-specific genetic backgrounds and environmental modifiers are likely to contribute to the observed epidemiological heterogeneity.

Most cases of MST are associated with syndromes, while only a small number of cases have been documented in individuals without recognised syndromes (Katanaki et al. 2025; Takahashi et al. 2017). Given that MST is a common finding in several syndromes (Lubinsky and Kantaputra 2016), dental practitioners may be the first healthcare providers to identify early manifestations of such conditions during routine clinical and radiographic examinations. For instance, cleidocranial dysplasia (Cano-Pérez et al. 2022), Gardner syndrome (Gupta et al. 2025), and Crouzon syndrome (Torun and Akbulut 2017) often involve MST, underscoring the importance of early identification. Dentists should therefore be trained to detect these conditions and correlate dental findings with other clinical and medical signs/symptoms reported by patients or caregivers. Recognising potential links between dental anomalies and systemic conditions is crucial, as these findings may collectively indicate an underlying syndrome (Giuca 2024). Although dentists are not expected to identify all known syndromes, they should develop a critical approach to assessing clusters of clinical signs/symptoms that may suggest a broader medical condition.

Data from this study indicate that individuals with MST are significantly more likely to experience root displacement of adjacent teeth due to the presence of ST. Such displacement can lead to adverse clinical outcomes, including diastema, crowding, root resorption, and unfavourable dental aesthetics (Anthonappa et al. 2013; Katanaki et al. 2025). Therefore, early detection plays a crucial role in preventing or minimising such complications. Management strategies for MST include surgical intervention, combined surgical and orthodontic treatment, or clinical follow-up alone. Early diagnosis benefits both patients and clinicians, as timely identification enables a broader range of treatment options, whereas delayed diagnosis may limit the available management modalities (Cortés-Bretón-Brinkmann et al. 2019; Ma et al. 2021; Maddalone et al. 2018; Seehra et al. 2023). However, the current body of evidence lacks standardised clinical guidelines. Moreover, robust systematic reviews, meta-analyses, and network meta-analyses are needed to assist in the determination of the most appropriate treatment strategies for different clinical scenarios involving SST or MST. Clinical decision-making should be guided by careful clinical and radiographic assessments, while also considering the preferences and values of patients and caregivers (Ata-Ali et al. 2014; Ma et al. 2021; Seehra et al. 2023). A widely accepted consensus suggests that surgical removal is preferable at an early stage of life. This approach is considered reasonable for several reasons, including the potential for more effective orthodontic management, when required, and the prevention of future complications (Cortés-Bretón-Brinkmann et al. 2019; Seehra et al. 2023).

The primary limitations of this study are inherent to its cross-sectional design (Wang and Cheng 2020), particularly the inability to establish causal relationships or assess long-term outcomes associated with SST or MST. The use of a convenience sample constitutes another important limitation, as it may introduce selection bias and restrict the generalisation of the findings to broader populations. The sample was derived from patients at a specialised paediatric oral surgery service, which may overrepresent individuals with more complex presentations or greater clinical needs. Moreover, it is important to emphasise that this study involved retrospective data collection and that all data were collected by a single examiner, despite the implementation of calibration procedures. Another limitation is the absence of follow-up data, particularly with regards to details on treatment and long-term outcomes. Despite these constraints, cross-sectional studies remain valuable for identifying associations and generating hypotheses that can be explored further using other study designs (Kesmodel 2018). In this context, it is plausible to hypothesise that SST and MST exhibit distinct patterns in terms of clinical characteristics, such as positioning, morphology, and eruption processes. Future research should address these aspects through population-based longitudinal studies, enabling the assessment of temporal relationships, disease progression, and the external validity of the findings.

## Conclusion

The findings of this study demonstrate that SST and MST have distinct clinical characteristics. MST were more frequently associated with supplemental morphology, palatal positioning, normal orientation, and a greater likelihood of eruption compared to SST. These results contribute to a more refined clinical understanding of supernumerary teeth and may assist clinicians in diagnostic reasoning and treatment planning. When this combination of features is identified during clinical or radiographic examinations, the possibility of MST should be considered. Clinicians should remain attentive to potential syndromic associations and carefully assess the need for intervention or structured follow-up, particularly in paediatric patients.

## Data Availability

No datasets were generated or analysed during the current study.

## References

[CR1] Adhikari M, Jha K, Shah A, Gurung M, Karmacharya K, Gurung S. Dentigerous cyst associated with impacted inverted mesiodens: a report of three cases and a brief literature review. Case Rep Dent. 2025;2025:6989796. 10.1155/crid/6989796. 40786705 10.1155/crid/6989796 PMC12335909

[CR2] Anthonappa RP, King NM, Rabie AB. Aetiology of supernumerary teeth: a literature review. Eur Arch Paediatr Dent. 2013;14(5):279–88. 10.1007/s40368-013-0082-z. 24068489 10.1007/s40368-013-0082-z

[CR3] Ata-Ali F, Ata-Ali J, Peñarrocha-Oltra D, Peñarrocha-Diago M. Prevalence, etiology, diagnosis, treatment and complications of supernumerary teeth. J Clin Exp Dent. 2014;4:e414–8. 10.4317/jced.51499. 10.4317/jced.51499 PMC4282911 25593666

[CR4] Azevedo SG, de Oliveira LQR, Martelli-Júnior H, Coletta RD, Machado RA. Tooth anomalies in patients with nonsyndromic orofacial cleft: a systematic review and meta-analysis. Oral Dis. 2025;31(7):2026–45. 10.1111/odi.15226. 39760181 10.1111/odi.15226

[CR5] Cammarata-Scalisi F, Avendaño A, Callea M. Main genetic entities associated with supernumerary teeth. Arch Argent Pediatr. 2018;116(6):437–44. 10.5546/aap.2018.eng.437. 30457727 10.5546/aap.2018.eng.437

[CR6] Cano-Pérez E, Gómez-Alegría C, Herrera FP, Gómez-Camargo D, Malambo-García D. Demographic, clinical, and radiological characteristics of cleidocranial dysplasia: a systematic review of cases reported in south America. Ann Med Surg (Lond). 2022;77:103611. 10.1016/j.amsu.2022.103611. 35638029 10.1016/j.amsu.2022.103611 PMC9142397

[CR7] Cassetta M, Altieri F, Giansanti M, Di-Giorgio R, Calasso S. Morphological and topographical characteristics of posterior supernumerary molar teeth: an epidemiological study on 25,186 subjects. Med Oral Patol Oral Cir Bucal. 2014;19(6):e545–9. 10.4317/medoral.19775. 25129242 10.4317/medoral.19775 PMC4259368

[CR8] Cortés-Bretón-Brinkmann J, Martínez-Rodríguez N, Barona-Dorado C, et al. Clinical repercussions and epidemiological considerations of supernumerary canines: a 26 case series. Med Oral Patol Oral Cir Bucal. 2019;24(5):e615–20. 10.4317/medoral.23035. 31422412 10.4317/medoral.23035 PMC6764710

[CR9] Davidson CL, Smit C, Nel S. Supernumerary teeth: a pictorial review and revised classification. J Oral Biol Craniofac Res. 2025;15(3):454–62. 10.1016/j.jobcr.2025.03.005. 40144646 10.1016/j.jobcr.2025.03.005 PMC11938152

[CR10] Fonseca-Souza G, de Oliveira LB, Wambier LM, Scariot R, Feltrin-Souza J. Tooth abnormalities associated with non-syndromic cleft lip and palate: systematic review and meta-analysis. Clin Oral Investig. 2022;26(8):5089–103. 10.1007/s00784-022-04540-8. 35729285 10.1007/s00784-022-04540-8

[CR11] Garvey MT, Barry HJ, Blake M. Supernumerary teeth–an overview of classification, diagnosis and management. J Can Dent Assoc. 1999;65(11):612–6. 10658390

[CR12] Giuca MR. Rare diseases: a challenge in paediatric dentistry. Eur J Paediatr Dent. 2024. 10.23804/ejpd.2024.25.03.01. 39212455 10.23804/ejpd.2024.25.03.01

[CR13] Gupta B, Chaudhari MA, Sohi HK, et al. Oral and maxillofacial manifestations of Gardner syndrome: a literature analysis. J Craniofac Surg. 2025. 10.1097/SCS.0000000000011639. 40623139 10.1097/SCS.0000000000011639

[CR14] Henninger E, Friedli L, Makrygiannakis MA, et al. Supernumerary tooth patterns in non-syndromic white European subjects. Dentistry J. 2023;11(10):230. 10.3390/dj11100230. 10.3390/dj11100230 PMC10605437 37886915

[CR15] Katanaki N, Makrygiannakis MA, Kaklamanos EG. The prevalence of supernumerary teeth in a sample of non-syndromic young patients from Greece. Dentistry J. 2025;13(7):317. 10.3390/dj13070317. 10.3390/dj13070317 PMC12293686 40710162

[CR16] Kesmodel US. Cross-sectional studies – what are they good for? Acta Obstet Gynecol Scand. 2018;97(4):388–93. 10.1111/aogs.13331. 29453895 10.1111/aogs.13331

[CR17] Kyeong M, Jeong JK, Adasooriya D, et al. Progressive tooth pattern changes in Cilk1-deficient mice depending on Hedgehog signaling. Int J Oral Sci. 2025;17(1):71. 10.1038/s41368-025-00405-4. 41320695 10.1038/s41368-025-00405-4 PMC12665794

[CR18] Liu F, Chu EY, Watt B, et al. Wnt/beta-catenin signaling directs multiple stages of tooth morphogenesis. Dev Biol. 2008;313(1):210–24. 10.1016/j.ydbio.2007.10.016. 18022614 10.1016/j.ydbio.2007.10.016 PMC2843623

[CR19] Lubinsky M, Kantaputra PN. Syndromes with supernumerary teeth. Am J Med Genet A. 2016;170(10):2611–6. 10.1002/ajmg.a.37763. 27250821 10.1002/ajmg.a.37763

[CR20] Ma X, Jiang Y, Ge H, et al. Epidemiological, clinical, radiographic characterization of non-syndromic supernumerary teeth in Chinese children and adolescents. Oral Dis. 2021;27(4):981–92. 10.1111/odi.13628. 32881166 10.1111/odi.13628

[CR21] Maddalone M, Rota E, Amosso E, Porcaro G, Mirabelli L. Evaluation of surgical options for supernumerary teeth in the anterior maxilla. Int J Clin Pediatr Dent. 2018;11(4):294–8. 10.5005/jp-journals-10005-1529. 30397373 10.5005/jp-journals-10005-1529 PMC6212659

[CR22] Sagai T, Amano T, Maeno A, et al. SHH signaling directed by two oral epithelium-specific enhancers controls tooth and oral development. Sci Rep. 2017;7(1):13004. 10.1038/s41598-017-12532-y. 29021530 10.1038/s41598-017-12532-y PMC5636896

[CR23] Seehra J, Mortaja K, Wazwaz F, Papageorgiou SN, Newton JT, Cobourne MT. Interventions to facilitate the successful eruption of impacted maxillary incisor teeth due to the presence of a supernumerary: a systematic review and meta-analysis. Am J Orthod Dentofacial Orthop. 2023;163(5):594–608. 10.1016/j.ajodo.2023.01.004. 36907703 10.1016/j.ajodo.2023.01.004

[CR24] Singh VP, Sharma A, Sharma S. Supernumerary teeth in Nepalese children. Sci World J. 2014;2014:215396. 10.1155/2014/215396. 10.1155/2014/215396 PMC4258912 25506609

[CR25] Takahashi M, Hosomichi K, Yamaguchi T, et al. Whole-exome sequencing analysis of supernumerary teeth occurrence in Japanese individuals. Hum Genome var. 2017;4:16046. 10.1038/hgv.2016.46. 28144447 10.1038/hgv.2016.46 PMC5267165

[CR26] Talaat DM, Hachim IY, Afifi MM, Talaat IM, ElKateb MA. Assessment of risk factors and molecular biomarkers in children with supernumerary teeth: a single-center study. BMC Oral Health. 2022;22(1):117. 10.1186/s12903-022-02151-z. 35397562 10.1186/s12903-022-02151-z PMC8994298

[CR27] Thomas E, Oettle AC, Becker PJ. Supernumerary teeth in a sample of South African dental patients. S Afr Dent J. 2023;78(2):74–81.

[CR28] Torun GS, Akbulut A. Crouzon syndrome with multiple supernumerary teeth. Niger J Clin Pract. 2017;20(2):261–3. 10.4103/1119-3077.187332. 28091449 10.4103/1119-3077.187332

[CR29] von Elm E, Altman DG, Egger M, Pocock SJ, Gøtzsche PC, Vandenbroucke JP, et al. The strengthening the reporting of observational studies in epidemiology (STROBE) statement: guidelines for reporting observational studies. J Clin Epidemiol. 2008. 10.1016/j.jclinepi.2007.11.008. 10.1016/j.jclinepi.2007.11.008 18313558

[CR30] Wang X, Cheng Z. Cross-sectional studies: strengths, weaknesses, and recommendations. Chest. 2020;158(1S):S65–71. 10.1016/j.chest.2020.03.012. 32658654 10.1016/j.chest.2020.03.012

